# Age-Related Changes in the Ability to Switch between Temporal and Spatial Attention

**DOI:** 10.3389/fnagi.2017.00028

**Published:** 2017-02-14

**Authors:** Eleanor Callaghan, Carol Holland, Klaus Kessler

**Affiliations:** ^1^Aston Research Centre for Healthy Ageing, Aston UniversityBirmingham, UK; ^2^Aston Brain Centre, Aston UniversityBirmingham, UK

**Keywords:** spatial attention, temporal attention, aging, switching, cognitive flexibility

## Abstract

**Background**: Identifying age-related changes in cognition that contribute towards reduced driving performance is important for the development of interventions to improve older adults’ driving and prolong the time that they can continue to drive. While driving, one is often required to switch from attending to events changing in time, to distribute attention spatially. Although there is extensive research into both spatial attention and temporal attention and how these change with age, the literature on switching between these modalities of attention is limited within any age group.

**Methods**: Age groups (21–30, 40–49, 50–59, 60–69 and 70+ years) were compared on their ability to switch between detecting a target in a rapid serial visual presentation (RSVP) stream and detecting a target in a visual search display. To manipulate the cost of switching, the target in the RSVP stream was either the first item in the stream (Target 1st), towards the end of the stream (Target Mid), or absent from the stream (Distractor Only). Visual search response times and accuracy were recorded. Target 1st trials behaved as no-switch trials, as attending to the remaining stream was not necessary. Target Mid and Distractor Only trials behaved as switch trials, as attending to the stream to the end was required.

**Results**: Visual search response times (RTs) were longer on “Target Mid” and “Distractor Only” trials in comparison to “Target 1st” trials, reflecting switch-costs. Larger switch-costs were found in both the 40–49 and 60–69 years group in comparison to the 21–30 years group when switching from the Target Mid condition.

**Discussion**: Findings warrant further exploration as to whether there are age-related changes in the ability to switch between these modalities of attention while driving. If older adults display poor performance when switching between temporal and spatial attention while driving, then the development of an intervention to preserve and improve this ability would be beneficial.

## Background

Driving cessation can be detrimental to older adults’ independence and has been shown to be a risk factor in developing depression (Marottoli et al., 1997; Ragland et al., 2005; Windsor et al., 2007). Identifying age-related changes in cognition that contribute towards reduced driving performance is the first step in a trajectory of research towards developing an intervention to improve older adults’ driving. This could lead to long-term advantages such as prolonging the time that people can continue to drive and help to preserve their independence.

At-fault collision statistics show that, while older adults have an overall reduced crash risk in comparison to young drivers, they present a disproportionate risk of at-fault collisions at intersections and collisions caused by a failure to give way, or to notice other objects, stop signs or signals (Hakamies-Blomqvist, 1993; McGwin and Brown, 1999; Guo et al., 2010; Arai and Arai, 2015). Consistent with higher risks at intersections (Hakamies-Blomqvist, 1993), in their seminal work Parasuraman and Nestor (1991) concluded that older drivers’ accidents were often due to failures in attention, particularly selective attention and switching. These findings are consistent with older drivers’ own self-perceptions, who have reported an increased difficulty to read and process signs in time (Musselwhite and Haddad, 2010). It is therefore a viable hypothesis that changes in spatial attention and attention switching are having an impact on driving skills later in life.

### Spatial Attention

There is extensive research demonstrating the relationship between spatial attention and driving performance and exploring how this changes with age (Hennessy, 1995; Richardson and Marottoli, 2003; Hoffman et al., 2005; Ball et al., 2006; Leversen et al., 2013; Cuenen et al., 2015). However, poor spatial attention does not result in poor driving in all older individuals (Vaucher et al., 2014). These findings highlight the need to further investigate attentional deficits in older drivers and identify the factors that determine whether deficits in attention affect driving performance.

There is a consensus that there is no specific decline in visual search performance with healthy aging when the target is distinct from the distractors and “pops out” of the display—i.e., a pop-out search (Plude and Doussardroosevelt, 1989; Foster et al., 1995; Humphrey and Kramer, 1997; Bennett et al., 2012; Li et al., 2013). Although older adults show increased response times (RTs) to detect targets in pop-out searches in comparison to young adults, age group differences in RTs remain constant with increasing numbers of distractors (Plude and Doussardroosevelt, 1989) and have therefore been attributed to general slowing (Foster et al., 1995). In contrast, visual search performance is thought to decline with age when the target is visually indistinct from distractors (i.e., in so-called “conjunction search”, where targets are defined as a combination of features shared with the distractors) and a serial search is required (Plude and Doussardroosevelt, 1989; Foster et al., 1995; Humphrey and Kramer, 1997; Bennett et al., 2012; Li et al., 2013). The increase in RTs with increasing numbers of distractors gets steeper with age, suggesting a specific deficit in serial visual search rather than a general slowing of RTs.

It is often argued that older adults have deficits in inhibiting irrelevant visual information (Hasher and Zacks, 1988; Greenwood and Parasuraman, 1994; Adamo et al., 2003; Maciokas and Crognale, 2003; Lustig et al., 2007; Gazzaley et al., 2008). It may be that poor selective attention is caused by deficits in inhibitory mechanisms. Competition models of visual selective attention (Treisman, 1988; Desimone, 1998; Bundesen et al., 2005; Beck and Kastner, 2009; Scalf et al., 2013) and evidence from single cell recordings (Reynolds et al., 1999) suggest that multiple stimuli are processed in parallel but in separate cell groups (Luck et al., 1997). Visual stimuli compete for processing resources and attention is implemented to bias excitation in favor of salient and task relevant stimuli. The Neural Theory of Visual Attention (NTVA; Bundesen et al., 2005) proposes that attention works to increase or decrease the number of cells involved in processing each object and alters the firing rate of neurons coding for certain features. Impairments in these excitatory-inhibitory attentional mechanisms may lead to difficulties in inhibiting irrelevant visual information and exciting target stimuli in older adults. This hypothesis would explain older participants’ disproportionately increased number and duration of saccadic eye movements on serial visual searches (Porter et al., 2010). In contrast, Lien et al. (2011) demonstrated that older and younger participants were equally able to attend to task-relevant stimuli and inhibit salient but irrelevant stimuli. However, it may be that the salience of the distractors aided inhibition due to the distinct visual features prompting a strong inhibitory response. Thus, deficits in excitatory-inhibitory mechanisms could lead to difficulties in selective attention.

There is evidence to suggest that older adults compensate for excitatory-inhibitory deficits with top-down control of attention. Neider and Kramer (2011) found that older participants not only benefited more than younger participants from using contextual information in a visual search within a realistic scene, but also displayed greater costs to their performance when the location of the visual search target was incongruent with its contextual information. Furthermore, McLaughlin and Murtha (2010) found that older adults utilized cues more than younger people in a visual search task. Similarly, Watson and Maylor (2002) demonstrated that the benefits of visual marking were preserved in adults aged 65–80 years. Visual marking is where a proportion of distractors within a visual search task is shown before the onset of the remaining distractor stimuli and the target stimulus, enabling top-down driven inhibition of the distractors that were presented first. However, the benefits of visual marking were not preserved in older adults when visual search items were moving. Previous research has demonstrated that older participants have lower motion detection thresholds (Conlon and Herkes, 2008) and find it more difficult to judge the speed of moving stimuli and vehicles (Scialfa et al., 1991; Schiff et al., 1992; Norman et al., 2003; Snowden and Kavanagh, 2006). The absence of visual marking of moving stimuli in older adults could therefore be due to difficulties in processing moving stimuli. Together findings suggest that older participants may rely more on top-down processes to compensate for declines in excitatory-inhibitory mechanisms in attention.

### Temporal Attention

In addition to the importance of spatial attention in driving, the allocation of attention to events changing in time, i.e., temporal attention, is important to be able to attend, process and respond to rapidly changing visual stimuli in a dynamic environment such as driving. It is well established that older adults require longer to process visual stimuli—i.e., have slower processing speeds (Ball et al., 2006; Rubin et al., 2007) and display an increased magnitude of the so-called “attentional blink” (Lahar et al., 2001; Maciokas and Crognale, 2003; Lee and Hsieh, 2009; Shih, 2009; van Leeuwen et al., 2009). The attentional blink is the reduced ability to detect a second target (T2) in a rapidly changing stream of stimuli—i.e., a rapid serial visual presentation (RSVP) stream—up to 500 ms after detecting a first target (T1) in the stream (Raymond et al., 1992). This effect is inflated and lasts for an increasing length of time with increased age. There is evidence to suggest that, whereas individuals in their 60s have no difficulties in temporal attention (Lee and Hsieh, 2009; Quigley et al., 2012), difficulties may begin to develop between the ages of 70–80 years (Conlon and Herkes, 2008; Shih, 2009). Conlon and Herkes (2008) concluded that impairments were due to slowed processing speed. However, age-related deficits observed in other temporal attention tasks have been demonstrated not to be due to general slowing (Maciokas and Crognale, 2003; Lee and Hsieh, 2009). Difficulties in temporal attention in those aged over 70 years could therefore be due to a specific decline in selective attentional mechanisms and could share the same underlying cause as difficulties in spatial selective attention, i.e., in excitatory-inhibitory selective attention processes, where excitatory mechanisms fail to respond to the target and inhibitory mechanisms fail to mitigate interference from the distractors in the RSVP stream.

### Switching Attention between Time and Space

Equally vital to safe driving, particularly at intersections, is the ability to switch between temporal and spatial attention. For example, when driving, one must switch from attending to fast moving and changing cars on the road ahead, to distributing attention across space to attend to road signs and surrounding hazards. Although there is extensive research displaying inflated switch-costs with increased age in task switching paradigms (Cepeda et al., 2001; Gamboz et al., 2009; Gold et al., 2010) there has been very little research on switching between different modalities of attention in any age group.

Overlapping networks across occipital, frontal, parietal and motor regions have been implicated in both directing attention in time and space (Coull and Nobre, 1998; Shapiro et al., 2002; Gross et al., 2004; Fu et al., 2005; Madden et al., 2007; Li et al., 2013). Although Coull and Nobre (1998) found overlapping activation for both temporal and spatial attention in a functional magnetic resonance imaging (fMRI) study, they found that patterns of activation for the two types of attention were distinct. In an extension of the NTVA, it has been proposed that as temporal expectation increases temporal attention works to increase the firing rate of neurons that represent certain features. In contrast, one would expect spatial attention to alter the number of cells allocated to processing objects in the visual field (Bundesen et al., 2005; Vangkilde et al., 2012, 2013). Thus, it may be expected that switching between temporal and spatial attention requires the efficient re-allocation of cells to receptive fields, as well as changes to the perceptual bias towards features which in turn influences the firing rate of neurons.

There is limited research into age-related changes in the ability to switch between temporal and spatial attention. Jefferies et al. (2015) demonstrated that younger adults require less time than older adults to narrow their focus of attention from two RSVP streams to one, indicating that there may be an age-related decline in the redistribution of attention spatially from a single location. However, both RSVP streams remained on the screen. Rather than a deficit in switching to distribute attention spatially, increased times taken to divert attention may be due to an age-related impairment in disengaging from task irrelevant stimuli (Greenwood and Parasuraman, 1994). In Lee and Hsieh (2009) study, participants switched from attending to an RSVP stream to identify a target, to allocating their attention in space to identify and point to a masked peripheral target in varying locations. Although the older age group displayed lower performance when the peripheral target was presented at 100, 300 and 700 ms after the RSVP target onset, lower performance was exaggerated at 100 and 300 ms. These findings show that older participants had greater difficulties in switching from temporal to spatial attention when there was 300 ms or less between target onsets. Russell et al. (2013) has since replicated these findings, further demonstrating that the impairment lasted for 450 ms. However, Lee and Hsieh (2009) aim was to investigate the attentional blink in older adults, resulting in a failure to distinguish between impaired task performance resulting from an increased attentional blink after processing the RSVP target, or due to increased switch-costs between temporal and spatial attention. Poorer performance at 100 and 300 ms, but not 700 ms, could equally be due to requiring longer to switch between temporal and spatial attention, or an extended attentional blink. A comparison of the relevant attentional blink and attention switching literature is presented in Table 1. The table compares the duration of the attentional blink in older age groups in addition to the duration of impairment from attention switching.

**Table 1:** **Comparison of results from previous studies**.

Author	Mean age (years)	Method	Duration of impairment
**Attentional blink**
Lahar et al. (2001)	68.70	Attentional blink	520 ms
Lee and Hsieh (2009)	59.30	Attentional blink	300 ms. No impairment at 700 ms
Maciokas and Crognale (2003)	64–79	Attentional blink	824 ms
**Switching**
Jefferies et al. (2015)	66.40	Time taken to narrow focus from 2 to 1 RSVP stream	266 ms
Lee and Hsieh (2009)	55–62	Attention switch from temporal to spatial attention	300 ms. No impairment at 700 ms
Russell et al. (2013)	66.00	Attention switch from temporal to spatial attention	450 ms

### The Current Study

The aim of the current study was to explore whether there are age-related changes in the ability to switch between temporal and spatial attention and to explore the cognitive mechanisms that might underpin these changes. Age groups were compared on their ability to switch from allocating attention in time, in order to identify a single target in an RSVP stream, to allocating attention spatially, in order to identify a visual search target. To manipulate the cost of switching, the position of the target in the RSVP stream was either the first item in the stream, towards the end of the stream, or absent from the stream. When the target was the first item in the stream (Target 1st condition), participants were no longer required to attend to the stream, and thus no cost of switching was expected. On the contrary, when the target was near the end of the stream (Target Mid condition) or the stream consisted of only distractor items (Distractor Only condition), participants needed to attend to the stream until towards the end of the stream, inducing a cost of switching. Longer visual search RTs were therefore expected when switching from the single target RSVP task to the visual search in both the Target Mid and Distractor Only conditions, which each behaved as switch conditions, in comparison to the Target 1st condition, which behaved as a no-switch condition. It was hypothesized that there would be an age-related increase in the cost of switching from the RSVP task to initiate the visual search task, which would be reflected in greater increases in RTs from the no-switch condition to the two switch conditions in the older groups in comparison to the younger groups. In contrast to Lee and Hsieh (2009) study, the inclusion of the Distractor Only condition enabled the investigation of whether any observed differences were due to difficulties in switching between attentional mechanisms or an increased attentional blink. If there is a deficit in switching between attentional mechanisms, then age-related inflated switch-costs would be present in both the Distractor Only and Target Mid conditions. Conversely, if age-related increases in switch-costs result from an extended attentional blink after processing the RSVP target, then age differences in switch-costs would only be observable in the Target Mid and not the Distractor Only condition.

Based on previous evidence that suggests that visual selective attention to temporal events is more difficult in those over the age of 70 years (Conlon and Herkes, 2008; Shih, 2009) but not in those aged 60–70 years (Lee and Hsieh, 2009; Quigley et al., 2012), it was expected that participants in the 70+ years age group would detect and identify fewer targets in the RSVP stream in comparison to younger adults, but that the 60–69 years age group would not be impaired.

It is well established that there is a decline in working memory capacity with increased age (Richardson and Vecchi, 2002; Toepper et al., 2014). It could be argued that the increased working memory load from retaining the target digit in the current task could impair older participants’ performance in switching. However, it is unlikely that retaining a single target would place enough demand on working memory to affect task performance. Furthermore, Akyürek and Hommel (2005) demonstrated that working memory load does not interact with the duration of the attentional blink, implying that working memory load should not affect visual search target processing. Although working memory capacity is unlikely to affect task performance in the current task, performance is likely to be affected by age-related declines to the central executive (Baddeley, 1992). Baddeley’s (1992) working memory model proposed that the central executive controls the allocation of attentional resources. It may therefore be expected that a decline in executive function could affect the ability to switch from allocating attention to events changing in time (i.e., the RSVP stream) to distribute attention spatially (i.e., to visual search stimuli). We therefore implemented the Random Number Generation task (RNG) to measure executive functions of updating and inhibition (Miyake et al., 2000) in order to examine the effect of executive function on task performance. Performance on random generation tasks has previously been found to decline with age (van der Linden et al., 1998).

Consistent with age-related declines in serial but not pop-out search performance (Plude and Doussardroosevelt, 1989; Foster et al., 1995; Humphrey and Kramer, 1997; Bennett et al., 2012; Li et al., 2013) and general slowing of RTs (Salthouse, 2000; Verhaeghen and Cerella, 2002), it was predicted that there would be an age-related increase in visual search RTs that would be greater for serial than pop-out searches.

To establish an understanding of the mechanisms that underpin switching between modalities of attention, additional cognitive measures were recorded. The useful field of view (UFOV) task was implemented to measure visual processing speed, divided attention and selective attention. Performance on the UFOV (Ball et al., 2006; Rubin et al., 2007; Edwards et al., 2009) tasks have been found to decline with age.

## Materials and Methods

### Participants

One hundred and five participants in five age groups (21–30, 40–49, 50–59, 60–69, and 70+ years) participated. The 21–30 years group were used as a comparison group for age-related cognitive changes for all other groups and the 40–49 and 50–59 years groups were used as middle-aged comparison groups for the 60–69 and 70+ years groups. Due to the study being advertized as research related to driving, many of the participants were regular drivers. The percentage of participants in each group who could drive are displayed next to participant demographics in Table 2. Participants with photosensitive epilepsy were excluded from participation, in addition to those who scored less than the 87 cut off for possible cognitive impairment on the Addenbrookes Cognitive Examination 3 (ACE-3; Noone, 2015). The ACE-3 consists of a series of short tasks which provide measures of language, memory, attention, fluency and visuospatial abilities.

**Table 2:** **Participant demographics**.

		Age group (years)				
		21–30 (*n* = 20)	40–49 (*n* = 20)	50–59 (*n* = 20)	60–69 (*n* = 21)	70+ (*n* = 21)
Age (years)	Mean	25.00	44.15	55.80	66.00	74.86
	SD	2.62	3.31	2.28	2.32	5.72
Gender	Male	10	9	8	10	8
	Female	10	11	12	11	13
Handedness	Right	19	18	17	21	19
	Left	1	2	2	0	2
	Ambidextrous	0	0	1	0	0
Level of education	A-Level	1	4	8	5	3
	Degree	4	7	8	10	9
	Post degree qualification	15	9	4	6	8
ACE-3	Mean	96.50	95.42	95.9	94.95	95.33
	SD	3.20	2.61	2.49	2.54	2.06
Drivers	Regular drivers	17	19	20	20	21

*The number of participants who are left and right handed, and the number of participants who are male and female are presented for each age group, in addition to the mean age of each age group. One participant from the 40–49 years group was excluded from the ACE-3 analysis as their performance was impaired on vocabulary dependent sections of the task due to English being their second language*.

Participants in the 21–30 and 40–49 years groups were recruited from Aston University staff and students and the community. Participants aged over 60 years were recruited from the Aston Research Centre for Healthy Ageing participation panel and University of the Third Age groups around the West Midlands. Participants received £7.50 towards their travel expenses. All participants provided written informed consent before participating. The research was approved by Aston University Research Ethics Committee. Vulnerable populations were not involved.

One participant from the 60–69 years group and two from the 70+ years group scored equal to or lower than the 87 cut-off on the ACE-3 (Noone, 2015) and were therefore excluded from further analyses. One participant in the 40–49 years group scored lower than 87 on the ACE-3, however, this was due to English being their second language and so they were not excluded from the study. Their ACE-3 score was excluded from the analysis. All other participants scored over 87. After excluding participants with low ACE-3 scores, the mean age of the 60–69 years group was 66.00 years (*SD* = 2.32), and the mean age for the 70+ years groups was 74.86 years (*SD* = 5.72).

Fisher’s Exact test comparing group differences in level of education (A-level equivalent or lower/degree equivalent/post degree qualification) revealed a significant difference in the level of education between groups (*p* = 0.049). The number of participants in the 21–30 years group with post degree qualifications was greater than expected (*z* = 2.1). A one-way ANOVA comparing group differences in general cognitive function measured with the ACE-3 revealed no significant group differences in ACE-3 scores (*p* > 0.05).

### Materials and Procedures

#### Attention Switching Task

In the attention switching task, participants alternated between attending to an RSVP stream and attending to a visual search display. Each trial consisted of a fixation cross, presented for 2000 ms, followed by the RSVP stream, which was immediately followed by the visual search display. Stimuli were presented on stimulus presentation software E-Prime 2.0 Professional (Psychology Software Tool. Inc., Sharsburg, PA, USA) on a windows computer, on a 22″ monitor (1280 × 1050 resolution) which was approximately 55 cm in front of the participant. All stimuli were presented in black (RGB 0-0-0) on a gray background (RGB 192-192-192).

The RSVP stream consisted of a rapidly changing stream of letters in the center of the display. There were ten items in each RSVP stream, each presented for 100 ms with no inter-stimulus interval. Stimuli were presented in font size 30 pt (0.75 × 0.75 cm, 0.78°). On two thirds of the trials, one of the items in the stream was a target digit ranging from 1 to 9. The participant’s task was to remember the digit. The remaining one third of the trials contained no target. Based on their visual similarity to certain numbers, letters I, O and S were excluded from the stream, as well as visual search targets K and Z. It should be noted that the current RSVP task differs from the attentional blink paradigm as the RSVP stream contains only a single target.

The visual search display consisted of eight letters presented in a circle around a fixation cross in the center of the screen, including seven distractors and one target. The target letter was always either a “K” or a “Z”. Stimuli were presented in font size 20 pt (0.50 × 0.50 cm, 0.52°) and the center of each stimulus was 2.3 cm (2.40°) from the center of the fixation cross. Participants pressed the “space-bar” once they identified the target. Participants’ RTs to press the space-bar were recorded. An initial space-bar response was implemented instead of a choice RT to minimize variability in RTs that result from the additional process of deciding which key to press. In a pilot study we found that while the overall pattern of means was the same, there was increased variability in RTs when participants responded by pressing either a “K” or “Z” key, depending on which letter the visual search target was, in comparison to using a single space-bar response. Higher variability from a choice RT may have affected older more than younger participants’ performance. Older participants are thought to show increased variability in RTs (Hultsch et al., 2002) and so it is important to minimize this variability. Participants were instructed to keep their eyes fixed on the cross while they completed the visual search and to respond as quickly as possible. Participants then indicated by typing on the keyboard whether it was a “K” or a “Z” in the display, followed by whether they had seen a target digit in the RSVP stream by typing “Y” if they had, and “N” if they had not. If a digit was correctly detected in the RSVP stream, participants then typed on the keyboard which number they saw. Accuracy throughout the task was recorded. Participants wore headphones through which a “ding” sound was played after a correct response and a chord sound was played after an incorrect response.

**Figure 1:**
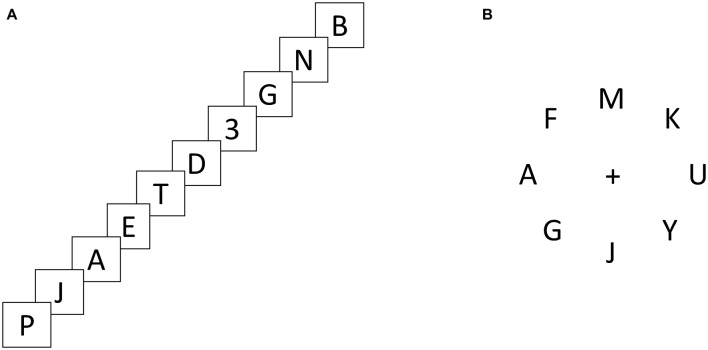
**Illustration of examples of the experiment set up.** The rapid serial visual presentation (RSVP) stream illustration **(A)** displays a Target Mid RSVP stream, and the visual search display illustration **(B)** displays a serial visual search display. Each trial consisted of a fixation cross (2000 ms) followed by an RSVP stream immediately followed by a visual search display.

On 50% of the trials the visual search display was a “pop-out” visual search, in which the distractors were all the letter “P”, allowing the target to “pop-out” to the participant. On 50% of the trials the visual search display was a “serial” visual search, in which all distractor letters were unique prompting a serial search. To manipulate the cost of switching, the position of the target in the RSVP stream that preceded the visual search was either the first item in the stream (Target 1st), which behaved as a no-switch condition, or the target was either the seventh, eighth or ninth item in the stream (Target Mid) or absent from the stream (Distractor Only), which both behaved as switch conditions. Illustrations of the RSVP stream and of the visual search display are presented in Figure 1.

There were 30 trials of each of the six conditions (Pop-out search: Target 1st/Target Mid/Distractor Only; Serial search: Target 1st/Target Mid/Distractor Only), with a total of 180 trials. To provide the opportunity for breaks, trials were divided into 10 blocks. Trials were randomized within blocks. Participants completed 10 practice trials before starting the experimental trials. One participant from the 40–49 years group, one from the 50–59 years group, five from the 60–69 years group and 11 from the 70+ years group required an additional 10 practice trials.

#### Useful Field of View Task

The Useful Field of View task (UFOV; Ball et al., 1988) was administered to measure processing speed, selective attention and divided attention. The UFOV consists of three sub-tasks on the computer, where the stimulus presentation duration begins at 500 ms and reduces to 16.7 ms until the participant achieves less than 75% accuracy. The shortest presentation duration at which the participant achieves 75% accuracy is recorded as the participant’s processing speed threshold in each of the tasks.

On the processing speed task, either a picture of a car or a picture of a truck was presented in the center of the screen. Participants then indicated whether the image presented to them was a car or a truck. The divided attention task was the same as the processing speed task with the addition of the simultaneous presentation of a peripheral stimulus, which was also either a car or a truck. Participants both identified the item presented in the center of the screen and the location of the peripheral stimulus. The selective attention task was the same as the divided attention task with the addition of distractor stimuli simultaneously presented surrounding the two target stimuli. A full description of the UFOV has been described previously by Ball et al. (1993).

#### Random Number Generation task

The RNG (Towes and Neil, 1998) was administered to measure executive functions. For 2 min, participants were played a metronome beat at 60 beats per minute and called aloud random numbers from 1 to 9 in time with the beat. Random was defined using Horne et al.’s (1982) hat analogy.

Towes and Neil (1998) software, Rgcalc, was used to calculate measures of randomness. In accordance with Miyake et al. (2000) Principal Components Analysis, Evans’ (1978) *RNG score*, a measure of how frequently number pairs/triplets occurred, was selected to measure inhibition, and *Redundancy* (R), a measure of how frequently each number occurred, was selected to measure updating. Lower scores on each measure indicates poorer randomization.

### Data Analysis

Data were analyzed using Statistical Package for Social Sciences (SPSS 21).

#### Attention Switching Task

Participants’ median visual search RTs (ms) on trials where responses were correct on both the visual search and RSVP tasks were extracted using E-Prime data viewing application E-DataAid. Participants’ proportions of correct visual search target identifications, RSVP target detections, RSVP target identifications and correct responses on the RSVP task on the Distractor Only condition were also extracted.

Older adults display higher proportions of false positive responses than younger individuals (Kenemans et al., 1995; Bolton and Staines, 2012). D prime (D′) was used as an unbiased measure of RSVP target detection sensitivity, as has been done in previous work on visual attention in the aging population (Parasuraman et al., 1989; Mouloua and Parasuraman, 1995; Berardi et al., 2001). Differences between age groups and RSVP conditions in both RSVP target detection and target identification were analyzed in two 2 × 5 mixed ANOVA, with RSVP condition (Target Mid/Target 1st) as a within subjects factor, and age group (21–30/40–49/50–59/60–69/70+ years) as a between subjects factor.

Differences in median visual search RTs between age groups, visual search conditions and RSVP conditions were analyzed in a 2 × 3 × 5 mixed ANOVA, where within subject factors were visual search condition (serial/pop-out) and RSVP condition (Distractor Only/Target Mid/Target 1st), and age group (21–30, 40–49, 50–59, 60–69, 70+ years) was a between subjects factor. Multiple comparisons were corrected for with Bonferroni correction.

The data were expected to violate assumptions of equality of variance due to increases in inter-individual variability with age (Hale et al., 1988; Morse, 1993). There is evidence to support that the ANOVA is robust to violations to homogeneity of variance (Budescu and Appelbaum, 1981; Budescu, 1982). Levene’s test for equality of variance is therefore not reported.

To further explore the interactions between independent variables that were identified from the ANOVA on RSVP accuracy, independent *t*-tests were implemented to compare age groups on target identification separately for Target Mid and Target 1st RSVP conditions.

To further explore the interactions between independent variables that were identified in the RT ANOVAs, percentage differences between conditions were calculated for each individual and independent *t*-tests were implemented to compare age groups’ percentage differences in RTs. It is important to note that *t*-tests were exploratory rather than hypothesis driven, and hence Restricted Fisher’s Least Significant Difference test was applied and corrections for multiple comparisons were not conducted (Snedecor and Cochran, 1967). Where Levene’s test for equality in variance was significant (*p* < 0.05) when computing *t*-tests, “Equality of variance not assumed” statistics were reported.

#### Cognitive Measures

The relationship between switch-costs and each cognitive measure, including UFOV subtasks processing speed, divided attention and selective attention and RNG indices for updating (R) and inhibition (RNG), and the relationship between each UFOV subtask and pop-out search RTs on the Target 1st condition, were explored with Spearman’s correlation analyses. It should be noted that correlations were exploratory and corrections for multiple comparisons were not conducted.

## Results

### Attention Switching Task

One participant in the 60–69 years group was excluded from the attention switching task analyses due to achieving chance level visual search accuracy in several conditions, including the Distractor Only pop-out search condition (mean = 0.40), the Target Mid pop-out search condition (mean = 0.53), and the Target Mid serial search condition (mean = 0.57). The participant’s low proportion of correct visual search responses indicates that they may not have understood the task. One participant from the 70+ years group was excluded from RT analyses due to poor visual search and RSVP target identification, resulting in less than one third of serial search trials remaining in the Target 1st condition. Nineteen participants remained in the 70+ years group in the RT analysis and there were 20 participants in 60–69 years group in the remaining analysis.

#### RSVP Accuracy

Both the ability to detect targets in the RSVP stream and the proportion of correctly identified targets, where the participant correctly reported the target digit, were examined. Poor target detection would suggest that participants have a deficit in temporal selective attention. Group differences in target identification and not target detection may indicate a deficit in consolidation or recall of the target. Thus, distinguishing between correctly detected and identified targets could reveal specific age-related deficits in different cognitive processes.

#### Target Detection

**Figure 2:**
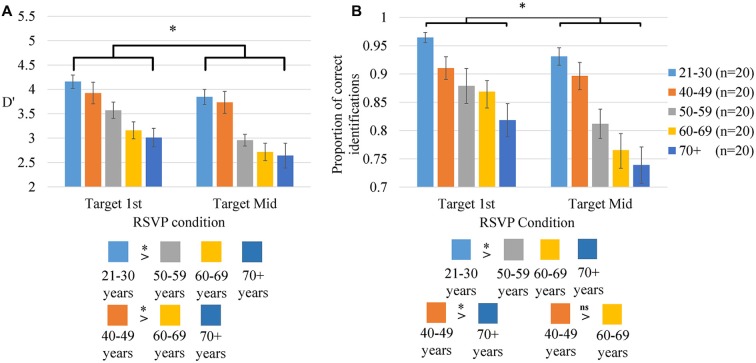
**RSVP Accuracy.** An index of RSVP target detection sensitivity, D′ **(A)** and the proportion of correctly identified RSVP targets **(B)** in each RSVP condition for each age group. The asterisk above each graph represents significant differences between RSVP conditions, collapsed across age groups. The color coded boxes below each graph illustrate significant age group differences collapsed across RSVP conditions. Vertical bars represent the standard error of the mean.

A 2 × 5 (RSVP condition × age group) ANOVA was conducted on measures of D′, an index of target detection sensitivity. D′ provides a measure of detection sensitivity while controlling for false positive response rates, which has been shown to be inflated in older participants (Kenemans et al., 1995; Bolton and Staines, 2012). D′ has previously been used as a measure of target sensitivity in work on visual attention in the ageing population (Parasuraman et al., 1989; Mouloua and Parasuraman, 1995; Berardi et al., 2001). D′ for each RSVP condition are presented for each age group in Figure 2.

There were significant main effects of age (*F*_(4,95)_ = 9.04, *p* < 0.001, $\eta_{\text{p}}^{2}$ = 0.28) and RSVP condition (*F*_(4,95)_ = 43.55, *p* < 0.001, $\eta_{\text{p}}^{2}$ = 0.31) on D′. There was no significant interaction between age and RSVP condition (*p* > 0.10).

##### Main effect of age

*Post hoc* comparisons revealed that the main effect of age on detection sensitivity resulted from greater detection sensitivity in the 21–30 years group in comparison to the 50–59 (*p* = 0.036), 60–69 (*p* < 0.001) and 70+ years (*p* < 0.001) groups. The 40–49 years group displayed a significantly higher detection sensitivity than the 60–69 (*p* = 0.005) and 70+ years groups (*p* = 0.001). There were no other significant group differences in detection sensitivity (*p >* 0.10).

No further analysis was carried out on D′. Age differences in target detection suggest that difficulties derive from declines in selective attention that will similarly affect RSVP target identification, as is evident in Figure 2. Instead, target identifications were examined in more depth.

#### Target Identification

Figure 2 illustrates a decrease in target identification with increased age. A 2 × 5 (RSVP condition × age group) mixed ANOVA was conducted on the proportion of correctly identified RSVP targets.

There was a significant main effect of age (*F*_(4,95)_ = 9.06, *p* < 0.001, $\eta_{\text{p}}^{2}$ = 0.28) and RSVP condition (*F*_(1,95)_ = 43.40, *p* < 0.001, $\eta_{\text{p}}^{2}$ = 0.31) on RSVP target identification, as well as a significant age × RSVP condition interaction (*F*_(4,95)_ = 3.15, *p* = 0.018, $\eta_{\text{p}}^{2}$ = 0.12).

##### Main effect of age

It was hypothesized that the 70+ years age group would identify fewer targets in comparison to younger groups but that there would be no difference in the proportion of targets identified in the 60–69 years age group. *Post hoc* comparisons showed that the main effect of age resulted from the 21–30 years group identifying significantly more RSVP targets than the 50–59 (*p* = 0.017), 60–69 (*p* = 0.001) and 70+ years (*p* < 0.001) groups. The 40–49 years group identified significantly more targets than the 70+ years group (*p* = 0.002). The higher target identification in the 40–49 years group in comparison to the 60–69 years group did not reach significance (*p* = 0.078). There were no other significant group differences in target identification (*p* > 0.10).

##### Main Effect of RSVP Condition

The main effect of RSVP condition resulted from participants identifying more targets in the Target 1st than the Target Mid condition.

##### Interaction between age and RSVP Conditions

To further explore the interaction between age group and RSVP condition on target identification independent *t*-tests were implemented to compare age groups on RSVP target identification on each RSVP condition separately.

In the Target 1st condition, the 21–30 years group identified significantly more targets than the 40–49 (*t*_(26.24)_ = 2.46, *p* = 0.021), 50–59 (*t*_(22.14)_ = 2.65, *p* = 0.015), 60–69 (*t*_(26.77)_ = 4.49, *p* < 0.001) and 70+ (*t*_(22.54)_ = 4.79, *p* < 0.001) years groups, and the 40–49 years group identified more targets than the 70+ years group (*t*_(38)_ = 2.60, *p* = 0.013).

In the Target Mid condition, the 21–30 years group identified significantly more targets than the 50–59 (*t*_(38)_ = 3.93, *p* < 0.001), 60–69 (*t*_(28.76)_ = 5.00, *p* < 0.001) and 70+ (*t*_(27.31)_ = 5.39, *p* < 0.001) years groups, the 40–49 years group identified significantly more targets than the 50–59 (*t*_(38)_ = 2.38, *p* = 0.022), 60–69 (*t*_(38)_ = 3.45, *p* = 0.001) and 70+ (*t*_(38)_ = 3.92, *p* < 0.001) years groups, and there was a non-significant trend for the 50–59 years group to identify more targets than the 70+ years group (*t*_(38)_ = 1.77, *p* = 0.086).

#### Visual Search

All groups correctly identified over 94% of visual search targets in all six conditions. Thus, no further analysis was carried out on visual search accuracy.

**Figure 3:**
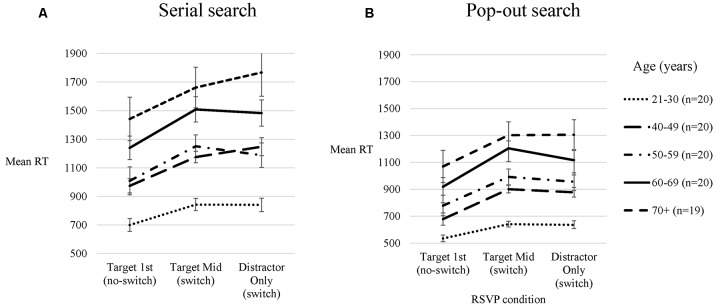
**Visual search response times (RTs).** Means of participants’ median visual search RTs for each RSVP condition for each age group on serial **(A)** and pop-out **(B)** visual searches. Vertical bars represent the standard error of the mean.

A 2 × 3 × 5 (visual search condition × RSVP condition × age group) mixed ANOVA was conducted with participants’ median RTs. Mauchly’s Test of Sphericity was significant for RSVP condition ($\chi_{\left(2\right)}^{2}$ = 8.56, *p* = 0.014) indicating that the assumption of sphericity has been violated. Greenhouse-Geisser corrected statistics were therefore reported. Mean visual search RTs for each age group for serial and pop-out searches are presented in Figure 3.

Significant main effects of age (*F*_(4,94)_ = 13.39, *p* < 0.001, $\eta_{\text{p}}^{2}$ = 0.36), visual search condition (*F*_(1,94)_ = 335.17, *p* < 0.001, $\eta_{\text{p}}^{2}$ = 0.78), and RSVP condition (*F*_(1.84, 172.80)_ = 133.57, *p* < 0.001, $\eta_{\text{p}}^{2}$ = 0.59) on visual search RTs were revealed, in addition to a significant age × visual search condition interaction (*F*_(4,94)_ = 4.98, *p* = 0.001, $\eta_{\text{p}}^{2}$ = 0.18), a significant age × RSVP condition interaction (*F*_(7.35, 172.80)_ = 2.72, *p* = 0.009, $\eta_{\text{p}}^{2}$ = 0.10), and a significant visual search condition × RSVP condition interaction (*F*_(1.99, 187.19)_ = 5.37, *p* = 0.005, $\eta_{\text{p}}^{2}$ = 0.05). There was no significant age × visual search condition × RSVP condition interaction (*p* > 0.10).

##### Main effects of age

Based on widely acknowledged age-related slowing of RTs, RTs were expected to increase with increased age (Salthouse, 1985, 2000; Foster et al., 1995). *Post hoc* comparisons illustrated that the 21–30 years group was significantly faster than the 50–59 (*p* = 0.024), 60–69 (*p* < 0.001) and 70+ (*p* < 0.001) years groups, but not the 40–49 years group (*p* > 0.10). The 70+ years group was slower than both the 40–49 (*p* = 0.001) and 50–59 years groups (*p* = 0.004). There were no other significant group differences in RT (*p* > 0.10).

##### Main effects of visual search condition

Participants were significantly faster on the pop-out in comparison to serial visual search.

##### Main effects of RSVP condition

We hypothesized that RTs would be faster on the no-switch (Target 1st) condition, when participants no longer need to attend to the RSVP stream after identifying the target digit, in comparison to when they are required to attend to the RSVP stream to the end of the stream in the two switch conditions (Target Mid/Distractor only). The main effect of RSVP condition on visual search RTs resulted from significantly faster RTs on the Target 1st condition in comparison to both the Distractor Only (*p* < 0.001) and Target Mid (*p* < 0.001) conditions. There was no significant difference between the Distractor Only and Target Mid conditions (*p* > 0.10).

##### Interaction between age and visual search conditions

It is well established that older participants display deficits in serial but not in pop-out visual searches (Plude and Doussardroosevelt, 1989; Foster et al., 1995; Humphrey and Kramer, 1997; Bennett et al., 2012; Li et al., 2013). It was hypothesized that the increase in RTs on serial in comparison to pop-out search would be greater in older than younger groups. In support of this hypothesis, there was a significant age × visual search condition interaction. To investigate this hypothesis further, the percentage increase in RTs from the pop-out to serial search, collapsed across RSVP conditions, was calculated and entered into independent *t*-tests to compare groups. To collapse visual search RTs across RSVP conditions separately for serial and pop-out search RTs, each participant’s median RTs was averaged across RSVP conditions separately for the pop-out search RTs and the serial search RTs. The mean percentage increase in RTs from pop-out to serial search is presented in Table 3.

**Table 3:** **Means and standard deviations of the percentage difference from Pop-out to Serial search RTs for each group**.

		Age Group (years)				
		21–30 (*n* = 20)	40–49 (*n* = 20)	50–59 (*n* = 19)	60–69 (*n* = 20)	70+ (*n* = 20)
Percentage	Mean	21.68	27.21	19.21	22.65	23.17
Difference	SD	11.01	7.61	9.95	8.41	8.58

*Percentage difference indicates the percentage increase in serial search RTs in comparison to pop-out search RTs*.

Independent *t*-tests revealed that there was a significantly larger difference between serial and pop-out search RTs in the 40–49 years than the 50–59 (*t*_(38)_ = 2.89, *p* = 0.007). The larger difference between pop-out and serial search RTs in the 40–49 years group in comparison to the 21–30 (*t*_(38)_ = −1.85, *p* = 0.072), 60–69 (*t*_(38)_ = 1.80, *p* = 0.080) and 70+ (*p* > 0.10) years groups did not reach significance. There were no further significant group differences in the percentage increase in RT from pop-out to serial search (*p* > 0.10).

##### Interaction between age and RSVP conditions

It was hypothesized that there would be greater difficulties in switching between the temporal and spatial attention tasks with increased age. To investigate the hypothesis that switch-costs would be greater with increased age, the interaction between age and RSVP condition was further explored. Each participant’s percentage increases in RTs from the no-switch (Target 1st) condition to each of the switch conditions (Target Mid/Distractor Only) were calculated as measures of switch-costs. Collapsing visual search conditions to calculate switch-costs lead to finding no significant age group differences in switch-costs (*p* > 0.10). Thus, although there was no three-way interaction between age, visual search condition and RSVP condition (*p* > 0.10), switch-costs were calculated separately for serial and pop-out search RTs to gain a detailed understanding of the interaction between age and RSVP conditions. The resulting measures of switch-costs were entered into independent *t*-tests to compare groups. It is important to note that *t*-tests were exploratory, however, remain in the scope of current hypotheses. The means and standard deviations of each group’s switch-costs on serial search and pop-out search RTs are presented in Table 4.

**Table 4:** **Means and standard deviations of switch-costs for each age group**.

			Age group (years)				
			21–30 (*n* = 20)	40–49 (*n* = 20)	50–59 (*n* = 19)	60–69 (*n* = 20)	70+ (*n* = 19)
Serial	Target mid	Mean	16.46	16.21	17.41	18.48	13.22
		SD	11.36	12.68	12.48	12.39	14.63
	Distractor	Mean	15.94	20.65	12.78	16.30	18.15
	Only	SD	13.26	10.85	21.23	14.33	12.68
Pop-out	Target mid	Mean	15.39	23.89	21.51	23.33	18.44
		SD	11.57	10.87	10.75	10.41	9.71
	Distractor	Mean	16.78	21.44	16.78	16.75	17.61
	Only	SD	8.48	10.01	15.03	13.28	11.84

*Switch-costs were calculated as the percentage increase in RT from the no-switch (Target 1st) condition to each of the switch conditions (Target Mid/Distractor Only) separately and separately for each visual search condition*.

The percentage increase in pop-out search RTs from the Target 1st to Target Mid condition were significantly greater for both the 40–49 (*t*_(38)_ = −2.39, *p* = 0.022) and 60–69 years groups in comparison to the 21–30 years group (*t*_(38)_ = −2.28, *p* = 0.028). The greater switch-costs in the 50–59 years group in comparison to the 21–30 years groups did not reach significance (*t*_(38)_ = −1.73, *p* = 0.091). There were no significant differences in switch-costs between any other age groups for either visual search condition (*p* > 0.10).

##### Interaction between visual search conditions and RSVP conditions

No further analysis was carried out on the interaction between RSVP condition and visual search condition, as it is unrelated to the current hypotheses.

### Cognitive Function

The cognitive mechanisms that underpin switching between modalities of attention were explored. Mean scores on UFOV processing speed, divided attention and selective attention, and the RNG index (inhibition) and R (updating) scores (Miyake et al., 2000) can be found in Table 5.

**Table 5:** **Means and standard deviations for cognitive measures**.

		Age group (years)				
		21–30 (*n* = 20)	40–49 (*n* = 20)	50–59 (*n* = 20)	60–69 (*n* = 21)	70+ (*n* = 21)
Updating (R)	Mean	1.41	1.45	1.06	1.22	1.08
	SD	1.78	1.07	0.81	0.52	0.71
Inhibition (RNG)	Mean	0.33	0.34	0.32	0.34	0.36
	SD	0.03	0.50	0.05	0.03	0.04
Processing speed	Mean	16.72	16.70	21.54	25.44	22.54
	SD	0.07	0.00	9.53	23.38	13.37
Divided attention	Mean	24.22	39.05	58.20	49.26	79.04
	SD	31.30	53.87	63.24	51.25	76.34
Selective attention	Mean	54.39	101.36	127.08	151.50	186.06
	SD	28.97	59.55	58.57	87.36	87.37

*One participant in the 70+ years group did not complete the UFOV, resulting in 20 participants in this group for the processing speed, divided attention and selective attention measures*.

#### The Relationship between Switch-Costs and Cognition

To identify cognitive functions that may affect switching ability, the relationships between switch-costs and cognitive measures were examined separately for each age group. Relationships were examined only for switch-costs in the target-switch pop-out search condition, as it was in this condition only that age group differences were found. Shapiro Wilks test of normality demonstrated that the distribution of scores from all cognitive measures except the RNG index violated the assumption of normality for one or more age groups (*p* < 0.05). Spearman’s rho correlation coefficients are therefore reported, which can be found in Table 6. Correlation strengths are interpreted based on Cohen (1992, 1988). It should be noted that correlations were exploratory and corrections for multiple comparisons were not conducted.

**Table 6:** **Spearman’s rho correlation coefficients, correlating cognitive measures with switch-costs**.

	Age group (years)				
	20–39 (*n* = 20)	40–49 (*n* = 20)	50–59 (*n* = 20)	60–69 (*n* = 20)	70+ (*n* = 19)
R	−0.03	−0.23	−0.03	0.13	−0.01
RNG	−0.16	−0.32	0.29	0.21	−0.14
Processing speed	−0.06	–	**−0.40**	−0.49*	0.14
Divided attention	0.22	0.04	−0.50*	0.07	−0.12
Selective attention	−0.17	0.12	−0.73***	**−0.43**	0.02

*p < 0.05, ***p < 0.001; Updating (R), inhibition (RNG).

#### UFOV Processing Speed

There was a significant negative moderate correlation between switch-costs and UFOV processing speed in the 60–69 years group (*p* = 0.033). Those with greater switch-costs displayed faster processing speeds. The correlation between switch-costs and processing speed did not reach significance in the 50–59 years group (*p* = 0.083). There were no other significant correlations between switch-costs and processing speed (*p* > 0.10).

#### UFOV Divided Attention

In the 50–59 years group there was a significant negative moderate correlation between switch-costs and UFOV divided attention (*p* = 0.027). Those with greater switch-costs performed better on the UFOV divided attention task (i.e., had faster processing thresholds). There were no other significant correlations between UFOV divided attention and switch-costs in any other age group (*p* > 0.10).

#### UFOV Selective Attention

There was a significant negative strong correlation between switch-costs and UFOV selective attention in the 50–59 years group (*p* < 0.001) and a non-significant negative moderate correlation between switch-costs and selective attention in the 60–69 years groups (*p* = 0.061). Participants with greater switch-costs had faster processing thresholds in the selective attention task. There were no other significant correlations between UFOV selective attention and switch-costs in any other age group (*p* > 0.10).

The direction of the relationship between switch-costs and performance on processing speed, divided attention and selective attention UFOV tasks was unexpected, as poor performance on the UFOV tasks was related to smaller switch-costs. These findings may be explained by a significant positive correlation between visual search RTs in the Target 1st condition and UFOV on processing speed (*r* = 0.445, *p* < 0.001, *n* = 99), divided attention (*r* = 0.592, *p* < 0.001, *n* = 99) and selective attention (*r* = 0.577, *p* < 0.001, *n* = 99). Those who perform poorly on the UFOV tasks have slower visual search RTs on the Target 1st condition. Slow RTs on the Target 1st condition result in smaller switch-costs, as the difference between switch and no-switch conditions becomes smaller.

#### RNG Task

There were no significant correlations between switch-costs and RNG measures R or RNG in any age group (*p* > 0.10).

## Discussion

The aim of the current study was to investigate whether there is an age-related decline in the ability to switch between temporal and spatial attention and to explore the cognitive mechanisms that might underpin these changes. Identifying age-related cognitive changes that affect driving behavior is an important first step in working towards developing a cognitive intervention to improve driving performance and prolong the length of time that people are able to continue to drive.

There were decreases in both RSVP target detection sensitivity and target identification with increased age. Deficits in target identification but not target detection would suggest that group differences are related to memory and not temporal attention. Results therefore indicate that older participants’ impaired performance derives from temporal attention mechanisms and results are not due to memory difficulties. It was hypothesized that there would be age-related difficulties in target identification in the 70+ years age group but not the 60–69 years age group. On the contrary, the 21–30 years group identified more targets than all other age groups in both the Target 1st condition and the Target Mid condition. Age group differences were more extensive in the Target Mid condition in comparison to the Target 1st condition, and significantly fewer targets were identified in the Target Mid condition in comparison to the Target 1st condition overall. Poorer target identification in the Target Mid condition likely results from the presence of distractor stimuli both forward and backward masking Target Mid targets, whereas Target 1st targets were only backward masked. It is likely that the effect of distractors masking the target was further exacerbated by older adults’ inhibitory deficits (Adamo et al., 2003; Maciokas and Crognale, 2003).

Consistent with previous research and with expectations, RTs were slower on serial than pop-out searches (Wolfe, 1998). These findings are due to attention being immediately drawn to the distinct target in pop-out searches, in contrast to when needing to complete a serial search (Treisman, 1985). Consistent with age-related slowing of RTs (Salthouse, 2000; Verhaeghen and Cerella, 2002), and supporting current hypotheses, there was an age-related increase in visual search RTs.

A greater increase in RTs from pop-out to serial search in the 40–49 years group in comparison to both younger and older groups was unexpected and contrasts with previous findings. Age-related deficits in serial but not pop-out search are well established (Plude and Doussardroosevelt, 1989; Foster et al., 1995; Humphrey and Kramer, 1997; Bennett et al., 2012; Li et al., 2013). The absence of greater differences between visual search conditions in the older groups may be due to ceiling effects, with slow RTs in both visual search conditions. In contrast, RTs on the pop-out search in the 40–49 years group remain fast and result in a larger percentage increase in RT from the pop-out to serial search.

Consistent with predictions, RTs were faster when switching from the RSVP task when the target was the first item in the stream in comparison to when the target was absent or in the middle of the stream. This can be attributed to larger switch-costs when attending to the RSVP stream to near the end of the stream than when able to disengage from the stream. These finding show that costs in switching from the temporal attentional task cause a delay to initiating a visual search.

Larger switch-costs in the 40–49 and 60–69 years groups in comparison to the youngest group for the Target Mid condition only partially supports the hypothesis that there would be increased switch-costs with age. Greater age-related switch-costs in only the 40–49 and 60–69 years age groups raises the question of why the 70+ years groups did not also display greater switch-costs in comparison to the youngest group and why the larger switch-costs in the 50–59 years group in comparison to the youngest group did not reach significance. RTs in the 40–49 years group were not significantly slower than RTs in the youngest group. It may be that fast RTs in the Target 1st condition are inflating the switch-costs in the 40–49 years group, as the percentage increase in RTs when they have to switch is greater. In contrast, switch-costs in the 50–59 years group are partially masked by their slow RTs in the Target 1st condition.

The question remains as to why greater switch-costs are seen in the 60–69 years and not in the 70+ years group. One explanation may be that the oldest group have developed efficient compensation strategies that are not yet present in the 60–69 years group. It may become necessary to adapt new strategies and recruit wider neural networks with older age due to increasingly impaired attentional and switching mechanisms combined with slowed RTs, whereas faster RTs in younger participants are sufficient to compensate for impaired switching. The recruitment of broader neural circuits with increased age is widely supported (Toepper et al., 2014), including in frontoparietal regions during attentional tasks (Madden et al., 2007), although it is unclear whether wider activation is due to compensation (Madden, 2007; Madden et al., 2007) or increased noise due to deficits in inhibitory mechanisms (Fabiani et al., 2006; Gazzaley et al., 2008). This raises the possibility that greater switch-costs were not seen in the 70+ years group due to increased variability in RTs masking switch-costs. Increased variability masking greater switch-costs in the 70+ years age group is supported by the increased variability observed with age in the current data.

A common limitation in aging research is self-selection bias. Older volunteers tend to be healthy, highly educated people who seek to stay active in later life. Both a physically, socially and cognitively active lifestyle, and higher levels of education and occupation have been shown to be protective factors against cognitive decline (Anstey and Christensen, 2000; Fratiglioni et al., 2004; López et al., 2014) and aspects of lifestyle such as level of education, video gaming habits and employment status have been shown to predict performance in visual attention tasks (Wilms and Nielsen, 2014). Thus, sample attributes may result in switch-costs in the 60–69 but not 70+ years, where there is less of a bias towards healthy, highly motivated people. However, the 70+ years group did not display a significantly higher level of education and did not perform better on the ACE-3, which is a basic measure of cognitive function.

As a third alternative, the difference between switch and no-switch conditions may have been reduced in the 70+ years group due to participants taking longer to process the Target 1st target and/or taking longer to disengage attention from the RSVP stream in the no-switch condition due to difficulties in inhibiting distractor stimuli. In both scenarios, visual search RTs in the no-switch condition would be inflated, reducing the difference between switch and no-switch conditions. These explanations would be consistent with increased visual processing speeds with increased age (Ball et al., 2006; Rubin et al., 2007) and with evidence that suggests that temporal attention is impaired only in those over the age of 70 years and not in those aged 60–69 years (Lee and Hsieh, 2009; Shih, 2009), explaining why increases in switch-costs are seen in the 60–69 years group and not the 70+ years group. This explanation would also account for the surprising findings of increased switch-costs with faster processing speed thresholds in the UFOV processing speed and selective attention tasks that were seen in both the 50–59 and 60–69 years groups. This relationship was in the opposite direction to expectations. To perform well on the UFOV selective attention task, one is required to inhibit irrelevant distractors across the screen to selectively attend to the target. Thus, inhibitory deficits seem to be resulting in both a smaller difference between no-switch and switch conditions, due to difficulties in disengaging from the RSVP stream, and longer processing speeds in the UFOV selective attention task. However, it is important to note that correlation analyses were exploratory and corrections for multiple comparisons were not conducted. Further research with larger sample sizes is needed to corroborate these findings.

However, switch-costs did not correlate with the RNG index, which is a measure of inhibition. This may be because inhibitory mechanisms implemented in the RNG task to inhibit repetition and number sequences are separate from those involved in inhibiting visual distractors. Excitatory-inhibitory competition in the visual cortex is involved in selectively attending visual information (Beck and Kastner, 2009; Reynolds et al., 1999), whereas inhibition during the RNG task is likely to involve the inhibition of response in working memory localized to the prefrontal cortex (Daniels et al., 2003). This conclusion is supported by Madden et al. (2007) who, in a serial visual search task, found that whereas young adults’ performance was associated with occipital lobe activation, older adults’ performance was more strongly related to frontal and parietal activity. These findings are consistent with a specific decline in serial search performance with age caused by deficits in excitatory-inhibitory mechanisms during visual processing.

Age differences in switch-costs in the Target Mid condition and not in the Distractor Only condition are likely due to the requirement to consolidate the RSVP target. It could be that increased switch-costs in this condition are due to slow processing speeds resulting in participants taking longer to process the target, which delays the switch to allocate attention spatially. On the contrary, fast processing speeds were related to increased switch-costs. Current findings therefore suggest that deficits in switching between temporal and spatial attention were not due to general slowing.

The current results support Lee and Hsieh (2009) findings of an age-related increase in difficulties in switching from attending to an RSVP stream to identify a target, to allocating attention in space to identify and point to a masked peripheral target. However, Lee and Hsieh (2009) aim was to investigate the attentional blink in older adults, and thus does not distinguish between impaired task performance resulting from an increased attentional blink, or due to deficits in switching between temporal and spatial attention. The inclusion of the Distractor Only condition in the current task enabled the investigation of whether age-related deficits in switching were due to increases in the time taken to switch between attentional mechanisms or an increased attentional blink. If there was a deficit in switching between attentional mechanisms, then older adults should be impaired in switching in both the Distractor Only and Target Mid conditions when compared with younger adults. Conversely, the current findings of increased switch-costs in the Target Mid condition only indicate that higher switch-costs may result from an increased attentional blink after processing the RSVP target. However, Figure 3 could also indicate that increased variability in the Distractor Only condition may have prevented group differences from emerging (e.g., 70+ group). Despite attempts to minimize variability in RTs by using an initial space-bar response, high variability masking differences in statistical power is corroborated by generally increased variability in RTs with increased age in the current dataset. The absence of a significant difference in RTs between the Distractor Only condition and the Target Mid condition further supports that group differences in switch-costs in the Distractor Only condition were not seen due to variability in RTs masking differences in statistical power.

Further work is required to explore how age-related declines in switching translate to driving behavior. It may be that difficulties in switching between temporal and spatial attention cause difficulties in switching from attending to traffic on the road ahead to attend to road signs and other surrounding objects. The current authors are presently exploring how attention switching predicts simulated driving performance. Previous work has shown that age-related difficulties in selective attention affect driving ability in some older drivers but not others (Vaucher et al., 2014). It may be that similarly, difficulties in switching between modalities of attention negatively affect the performance of some drivers but not others. If difficulties in switching are found to affect driving performance, then it will be important to develop an intervention to improve switching between modalities of attention to help improve driver performance and safety. Long-term, this will prolong the time that older drivers can continue to drive and help to preserve their independence.

### Limitations

In contrast to previous visual search paradigms (Humphrey and Kramer, 1997; Li et al., 2013), participants were required to make an initial space-bar response to indicate that they had identified the target and then report which letter they had seen. A limitation of this approach is that participants may modify their decision after they have made a response with the aid of visual memory. It is not known whether the ability to adopt this strategy is greater in young adults than older adults. However, the opportunity to implement this strategy was present in both switch and no-switch trials and therefore should not have affected our main findings.

A further limitation of the current paradigm is that we did not explore how switching affects the ongoing visual search processes, as the number of distractor stimuli in the visual search display was not manipulated. In the current paradigm we were interested in the efficiency of switching to initiate a search. Further research is required to investigate how switching influences ongoing search processes. It may be that switching has a large effect on search speed at the beginning of a search but the effect on search speed plateaus with increasing numbers of distractors, as time since the switch increases. Furthermore, it may be that this switch-cost is not specific to switching between types of attention, but would also affect performance in other cognitive functions. Although it is switching between temporal and spatial attention that is important to driving, where declines in efficiency may have a negative impact on a person’s life, this difficulty may generalize to other tasks. This is an important question to ask when developing an intervention.

The 40–49 and 50–59 years age groups were intended as middle-age comparison groups for the two oldest age groups and the 21–30 years group was intended as a comparison group for all other age groups. The finding of higher switch-costs in the 40–49 years group was unexpected, particularly as no differences in RTs were found between the 21–30 and 40–49 years groups. Future research would benefit from also including a 30–39 years age group in order to obtain a view of how the ability to switch between attentional mechanisms changes throughout the adult lifespan.

It is well established that working memory capacity declines with healthy aging, including both verbal (Hultsch et al., 1992; Zacks et al., 2000) and visual (Faubert, 2002; Brockmole and Logie, 2013) short term memory. A limitation of the current study is that no measure of verbal or visual working memory capacity was taken to look at the influence of memory on switching. However, the strain on working memory is very low, as the participant is only required to hold a single item in memory (i.e., the RSVP target digit). It is therefore unlikely that difficulties in working memory would affect switching performance. Furthermore, Akyürek and Hommel (2005) found that working memory load did not interact with the duration of the attentional blink. Additionally, working memory load remains constant across both the no-switch and Target Mid switch conditions and so memory deficits should not have influenced our main findings.

Although age-related declines in working memory capacity are unlikely to have affected switching performance in the current task, it is possible that declines in executive function affected switching performance. In relation to Baddeley’s (1992) working memory model, the current task would require the top-down control of attention from the central executive. It was therefore expected that measures of executive function would predict switching performance. However, measures of executive function obtained from the RNG did not correlate with task performance. It was found that there were no age group differences in RNG measures, despite age-related declines in executive function being widely acknowledged in the literature (Cepeda et al., 2001; Gamboz et al., 2009; Gold et al., 2010). It may be that RNG performance is too susceptible to interference from the use of alternative strategies, such as visualization techniques. Further research is needed to explore the relationship between executive function and switching between temporal and spatial attention to come to more sound conclusions.

A further limitation of the methodology is that eye tracking data were not recorded and participants’ actual fixation was not controlled for to ensure that participants were focusing on the visual search fixation cross. Participants’ failure to focus on the fixation cross could result in error in the measurements of RTs to complete the visual search. However, participants were instructed to keep their eyes fixed on the fixation cross, a protocol that is commonly used across cognitive paradigms (Humphrey and Kramer, 1997; Watson and Maylor, 2002; Li et al., 2013). Furthermore, participants’ attention to the RSVP stream before the onset of the visual search display ensured that participants were focusing on the center of the screen at the beginning of each trial. Trials in which participants failed to correctly identify the target digit in the RSVP stream were excluded from RT analyses, which ensured that only trials in which participants were attending to the task were included in analyses.

Gender differences in the decline of certain attentional mechanisms have previously been found (Conlon and Herkes, 2008). The current study did not look at gender differences in age-related changes in switching ability as it was beyond the scope of the study. Future work could investigate whether there are any gender differences in the ability to switch between temporal and spatial attention in older age.

### Conclusions and Future Directions

The hypothesis that there would be greater switch-costs in older than younger groups was partially supported, as people aged 40–49 and 60–69 years displayed greater switch-costs than those aged 20–29 years. There was also a non-significant trend for greater switch-costs in the 50–59 years group. Increased switch-costs in the 40–49 and 60–69 years groups but not the 70+ years groups was surprising. However, switching difficulties in the oldest group may have been masked by slow RTs on the Target 1st condition due to a failure to inhibit and disengage from the RSVP stream. This conclusion would explain the surprising findings of decreased switch-costs with slower selective attention processing speeds. Poor selective attention could mask switch-costs due to difficulties with inhibiting the remainder of the RSVP stream in the Target 1st condition resulting in slow RTs. Future studies investigating switching between temporal and spatial attention would benefit from including a condition that contains a target with no distractor stimuli.

Increased switch-costs in the Target Mid condition but not the Distractor Only condition indicates that increased switch-costs could result from either an increased attentional blink following RSVP target identification, which delays the allocation of attentional resources to the visual search, or increased variability in RTs in the Distractor Only condition.

The current authors are presently investigating whether age-related difficulties in switching affect driving performance. If difficulties in switching affect driving performance, then this cognitive process should be targeted in the development of interventions that aim to improve driver performance and safety. Long-term, this will prolong the time that older drivers can continue to drive.

## Author Contributions

EC contributed towards the design of the research, played a key role in data collection, data analysis and interpretation of the analysis, in addition to drafting and revising the written article and approving the final version to be published. CH contributed towards the conception and design of the work, to the data analysis and interpretation, in addition to contributing towards the critical revision of the article and approving the final version to be published. KK made a substantial contribution to the conception and design of the work, to the data analysis and interpretation, in addition to contributing towards the critical revision of the article and approving the final version to be published.

## Conflict of Interest Statement

The authors declare that the research was conducted in the absence of any commercial or financial relationships that could be construed as a potential conflict of interest. The reviewer DW declared a collaboration with one of the authors CH to the handling Editor, who ensured that the process met the standards of a fair and objective review.
